# Stroke Awareness and Knowledge Gaps in Saudi Arabia: A Cross-Sectional Study

**DOI:** 10.7759/cureus.104227

**Published:** 2026-02-25

**Authors:** Shorog M Althubait, Mohamed H Sarhan, Omar A Alotaibi, Leen S Alsobhi, Turki D Albeladi, Doha A Alabdulwahab, Sarah M Al-Bishr, Waad R Alghamdi, Sultan S Sifran, Huda Y Alhashem, Rehab E Alhazmi, Emad F Al-Sulami, Norah M Alwadai, Khames Alzahrani

**Affiliations:** 1 Department of Neurology, College of Medicine, King Khalid University, Abha, SAU; 2 Department of Basic Medical Sciences, College of Medicine, Shaqra University, Shaqra, SAU; 3 Department of Medical Parasitology, Faculty of Medicine, Zagazig University, Zagazig, EGY; 4 Department of Medicine, College of Medicine at Dawadmi, Shaqra University, Dawadmi, SAU; 5 Department of Medicine, Faculty of Medicine, King Abdulaziz University, Jeddah, SAU; 6 Department of Medicine, Ministry of Health, Jeddah, SAU; 7 Department of Medicine, College of Medicine, Najran University, Najran, SAU; 8 Department of Medicine, Vision Colleges, Riyadh, SAU; 9 Department of Medicine, Ibn Sina National College for Medical Studies, Jeddah, SAU; 10 Department of Medicine, Faculty of Medicine, University of Jeddah, Jeddah, SAU; 11 Department of Medicine, Ministry of Health, Riyadh, SAU

**Keywords:** complications, health education, public awareness, risk factors, saudi arabia, stroke, warning signs

## Abstract

Background

Stroke is a leading cause of death and disability, yet public awareness of stroke remains insufficient. This study aimed to assess the awareness of stroke among the general Saudi population.

Methodology

A cross-sectional study was conducted using an online self-administered questionnaire targeting Saudi residents aged 18 years and older. The survey evaluated general stroke knowledge, recognition of warning signs, and understanding of potential complications.

Results

A total of 938 participants completed the survey, with a mean age of 28.53 years; 65.2% were female. Only 9% of the participants demonstrated high general awareness of stroke, while 61.4% scored low. Awareness of stroke as a medical emergency was high (92.6%), yet knowledge of complications was limited, with 67.5% scoring low. Female and single participants showed significantly higher knowledge levels (p < 0.05), and higher education was strongly associated with better awareness (p < 0.001).

Conclusions

The study revealed substantial gaps in stroke-related knowledge among the Saudi population, particularly among males, married individuals, and those with lower educational attainment. Targeted public health interventions are needed to improve awareness, focusing on culturally appropriate education that emphasizes early symptom recognition and risk factor management.

## Introduction

The outcome of stroke is strongly affected by timely access to acute medical interventions, particularly thrombolytic therapy. Early identification of stroke warning signs is crucial to ensure prompt utilization of these critical services, leading to improved outcomes [1]. Yet, numerous reports have consistently demonstrated that public knowledge regarding risk factors and warning indicators of stroke is still unsatisfactory. Alarmingly, the unsatisfactory awareness is often obvious among populations at highest risk, notably individuals over the age of 75. Furthermore, even among those who acknowledge having stroke-related risk factors, knowledge of early stroke symptoms does not appear to be significantly better than that found in individuals who do not have such risk factors [2-4].

Stroke is considered the most preventable neurological disorder. Most well-known risk factors, such as diabetes mellitus, hypertension, hyperlipidemia, smoking, and cardiovascular disease, can be effectively controlled with lifestyle modifications and/or pharmacological treatment [3,5]. Hence, enhancing public understanding about perceived risk factors and early warning signals of stroke is crucial for guiding preventive health strategies aimed at reducing stroke-related outcomes. Importantly, such interventions need not be complex; even simple educational initiatives lead to better impact on stroke prevention and early recognition with favorable outcomes [3,6,7].

The need to raise public awareness of stroke risk factors and warning signs has been identified as crucial to addressing the significant knowledge gaps. Population-based research shows that people are not fully aware of stroke risk factors and warning symptoms of stroke onset. Patients interviewed after being admitted to the hospital for a stroke revealed similar findings. Current European data on stroke awareness in the general community are sparse [8].

In Arabic countries, population-based studies have demonstrated unsatisfactory levels of awareness regarding both modifiable risk factors and the early symptoms indicative of stroke onset [9,10]. Over the last two decades, environmental and lifestyle changes in Saudi Arabia have increased the risk and incidence of stroke [11]. Yet, studies evaluating knowledge of the general population about stroke are scarce [12,13]. Hence, we conducted this study to assess the knowledge and awareness level of stroke risk factors, symptoms, and complications among the Saudi population because of the general population’s low and satisfactory level of awareness about stroke and its preventable risk factors, in addition to the paucity of literature on such issues in Saudi Arabia.

## Materials and methods

Study design

A two-month, online, observational, cross-sectional study was conducted across Saudi Arabia from June 1 to July 31, 2025. The authors designed a systematic questionnaire to collect data on the general population’s awareness and understanding of stroke and its risk factors.

Selection criteria

Participants 18 years or older, male or female, living in Saudi Arabia, and who provided their approval for the study were considered for inclusion in the study. Individuals under the age of 18 years, those living outside of Saudi Arabia, those who refused to participate, and those who submitted incomplete questionnaires were excluded.

Ethical considerations

The Research Ethics Committee of King Khalid University, Saudi Arabia (HAPO-06-B-001) provided institutional review board approval (approval reference: KKU-4-2025-9; approval date: 2025-04-29). The study adhered to the Helsinki Declaration’s ethical criteria. Participation was entirely voluntary, and replies remained anonymous. Before accessing the survey, all participants provided written informed consent (online consent) to participate in the study.

Pilot test

Pilot testing was conducted among 50 individuals, and the results were not used in the final analysis. Participants self-administered the survey, which was provided in Arabic, the native language of Saudi Arabia. At the outset of the survey, participants were provided a written Participant Information Statement that described the study’s principal purpose and the estimated time required to complete the questionnaire (5-10 minutes).

Sample size

The sample size was determined using the Cochran formula. We used a 95% confidence level, a 50% response rate (0.5), and a 0.05 confidence interval (±5). Sample size was calculated using the following formula: sample size (n) = (Z-score)^2^ × P × (1 - P)/(margin of error)^2^ = ([1.96]^2^ × 0.5 [0.5])/(0.05)^2^ = 384.16. The expected sample size was 384. A total of 1,019 participants were invited to participate in the survey. Overall, 938 (92.1%) individuals accepted and completed the questionnaire, while the others were excluded, where 17 (1.7%) subjects refused to participate, and 64 (6.3%) subjects were non-Saudi residents.

Structure of the questionnaire

We used an online self-administered Arabic/English questionnaire (Appendices). Data for this study were collected using a structured, self-completed online survey (X (formerly Twitter), WhatsApp, and Facebook), created by the authors by reviewing previous literature that used a set of questions to accurately capture multiple aspects of stroke knowledge in various populations [12,14].

The questionnaire was divided into five main components (Appendices). The first section had eight questions about socioeconomic background. The second section included knowledge questions about stroke, such as the definition and meaning of stroke (22 questions). The third section evaluated the level of stroke severity and how to act (three questions). The fourth section included questions regarding the awareness of stroke symptoms (10 questions). The fifth section contained questions about complications (seven questions).

Scoring system

Zero point was given for incorrect answers or “I don’t know.” For scoring, we utilized Likert scales (dichotomous, three-point, and quality scales). The maximum score was 42 and was divided as follows: the original Bloom cut-off values were 80.0%-100%, 60.0%-79%, and 59.0%. We sorted the participants into three categories according to their scores.

Knowledge scores ranged from 0 to 22 and were divided into three categories: low level of knowledge with a score of 12 or <13, moderate level of knowledge with a score between 14 and 16, and high level of knowledge with scores of 17 or above.

Awareness scores ranged from 0 to 3 points and were divided into three categories: low level (score 1 or below), moderate level (score 2), and high level (score 3).

The knowledge of warning signs was graded on a scale of 0 to 10, with three categories: low level (4 or fewer than 5 points), moderate level (6 to 7 points), and high level (8 points or more).

The knowledge of complications of stroke scores ranged from 0 to 7 points, with three levels: low (4 points or lower), moderate (5 points), and high (6 points or more).

Statistical analysis

We used descriptive statistics to describe sociodemographic characteristics and assess general knowledge, risk factors, early symptoms, and consequences identified by participants. We presented continuous variables as mean and standard deviation (SD), whereas categorical variables were presented as frequencies (n) and percentages (%). Student’s t-test and chi-square test were used for continuous and nominal data, respectively. Meanwhile, we used logistic regression analysis to identify different predictors that affect the level of stroke knowledge and awareness, as well as its risk factors, symptoms, and complications. The level of confidence was set at 95%, and a p-value <0.05 was considered significant.

## Results

Participant characteristics

The enrolled individuals’ average age was 28.53 years. Most subjects were males (n = 326, 65.2%) and single (n = 616, 65.7%). Additionally, a total of 711 (75.8%) participants had a university-level education or higher. A total of 362 (38.6%) subjects had a monthly income <1,000 (SAR). Only 20 (2.1%) subjects had a previous history of stroke (Table 1).

**Table 1:** Characteristics of enrolled subjects. Data are presented as mean (SD) and frequency (percentage).

Item	Analysis (n = 938)
Age (years)	28.53 ± 10.76
Sex	
Male	326 (34.8%)
Female	612 (65.2%)
Region	
Eastern	85 (9.1%)
Middle	197 (21%)
Northen	102 (10.9%)
Southern	168 (17.9%)
Western	386 (41.2%)
Marital status	
Married	305 (32.5%)
Divorced	16 (1.7%)
Widowed	1 (0.10%)
Single	616 (65.7%)
Education level	
Primary	5 (0.50%)
Secondary	10 (1.1%)
High school	212 (22.6%)
University and above	711 (75.8%)
Monthly income (SAR)	
<1,000	362 (38.6%)
1,000–5,000	225 (24%)
5,001–10,000	115 (12.3%)
10,001–15,000	113 (12%)
>15,000	123 (13.1%)
Have you ever had a stroke before?	20 (2.1%)

Correct answers and the level of knowledge and awareness about stroke

A total of 514 (54.8%) subjects considered that symptoms of stork occurred suddenly. The average level of general knowledge was 11.80 ± 3.90. Overall, 576 (61.4%), 278 (29.6%), and 84 (9%) participants had low, mild, and high levels, respectively.

In total, 872 (93%) and 869 (92.6%) participants thought that stroke was one of the leading causes of death and considered it a medical emergency, respectively. Furthermore, 895 (95.4%) participants considered that behavior change occurred with stroke. Overall, 820 (87.4%), 78 (8.3%), and 40 (4.3%) participants had high, moderate, and low levels of awareness.

Regarding the level of knowledge about alarm signs of stroke, 548 (58.4%), 245 (26.1%), and 145 (15.5%) participants had low, mild, and high levels of knowledge, respectively. Regarding the level of knowledge about complications, 633 (67.5%), 212 (22.6%), and 93 (9.9%) participants had low, mild, and high levels of knowledge, respectively (Table 2).

**Table 2:** Correct answers and level of knowledge and awareness about stroke. Data are presented as mean (SD) and frequency (percentage).

Item	Analysis (n = 938)
General knowledge	
Have you ever heard about strokes?	864 (92.1%)
What causes a stroke?	717 (76.4%)
Do you think there are other names for stroke?	509 (54.3%)
Is hypoglycemia a risk factor for stroke?	185 (19.7%)
Are diabetic patients at risk of stroke?	522 (55.7%)
Is iron deficiency a risk factor for stroke?	495 (52.8%)
Do you think high blood pressure is a risk factor for stroke?	756 (80.6%)
Do you think high levels of cholesterol are a risk factor for stroke?	625 (66.6%)
Is epilepsy a risk factor for stroke?	127 (13.5%)
Do you think heart disease is a risk factor for stroke?	644 (66.6%)
Do you think Alzheimer’s disease is a risk factor for stroke?	279 (29.7%)
Can family history cause a stroke?	572 (61%)
Is excessive sun exposure a risk factor for stroke?	362 (38.6%)
The incidence of stroke increases at 65 years and older	266 (28.4%)
Do you think lack of exercise is a risk factor for stroke?	628 (67%)
Do you think obesity or being overweight is a risk factor for stroke?	615 (65.6%)
Do you think an unhealthy diet is a risk factor for stroke?	156 (16.6%)
Do you know the symptoms of stroke that usually appear?	514 (54.8%)
Do you think smoking is a risk factor for stroke?	669 (71.3%)
Do you think stress is a risk factor for stroke?	736 (78.5%)
Do you think atrial fibrillation is a stroke risk factor?	576 (61.4%)
Do you think that it is possible to cure a stroke when arriving at the hospital early?	611 (65.1%)
Level of knowledge	
Mean ± SD	11.80 ± 3.90
Low	576 (61.4%)
Mild	278 (29.6%)
High	84 (9%)
Awareness about stroke	
Do you think stroke is one of the leading causes of death?	872 (93%)
Is a stroke a medical emergency?	869 (92.6%)
Does behavior change with a suspected stroke?	895 (95.4%)
Level of awareness	
Mean ± SD	2.81 ± 0.57
Low	40 (4.3%)
Mild	78 (8.3%)
High	820 (87.4%)
Knowledge of alarm signs	
Do you think sudden confusion, trouble speaking, or difficulty understanding speech is a stroke symptom?	670 (71.4%)
Do you think a sudden nosebleed is a stroke symptom?	260 (27.7%)
Do you think sudden numbness or weakness of the face, arm, or leg is a symptom of stroke?	647 (69%)
Is sudden trouble seeing in one or both eyes a stroke symptom?	560 (59.7%)
Do you think sudden vomiting is a stroke symptom?	268 (28.6%)
Do you think back pain is a stroke symptom?	427 (45.5%)
Do you think chest pain is a stroke symptom?	326 (34.8%)
Do you think a sudden, severe headache with no known cause is a stroke symptom?	552 (58.8%)
Do you think sudden trouble walking, dizziness, loss of balance, or coordination are stroke symptoms?	633 (67.5%)
Do you think a high temperature is a symptom of a stroke?	300 (32%)
Level of knowledge of alarm signs	
Mean ± SD	4.95 ± 2.50
Low	548 (58.4%)
Mild	245 (26.1%)
High	145 (15.5%)
Knowledge of complications	
Do you think cerebral edema is a complication of stroke?	577 (61.5%)
Do you think pneumonia is a complication of a stroke?	249 (26.5%)
Do you think paralysis is a complication of a stroke?	639 (68.1%)
Do you think memory loss is a complication of a stroke?	622 (66.3%)
Do you think feeling depressed and anxious is a complication of a stroke?	409 (43.6%)
Do you think kidney disease is a complication of stroke?	333 (35.5%)
Do you think ulcerative colitis is a complication of stroke?	370 (39.4%)
Level of knowledge of complications	
Mean ± SD	3.41 ± 1.84
Low	633 (67.5%)
Mild	212 (22.6%)
High	93 (9.9%)

Factors affecting the knowledge of participants about stroke

Sex and marital status were found to significantly affect the level of general knowledge, with female and single subjects having a high level of knowledge (Table 3).

**Table 3:** Factors affecting knowledge of participants about stroke. *: P-value is significant at <0.05. Data are presented as mean (SD) and frequency (percentage).

Item	Low (n = 576)	Mild (n = 278)	High (n = 84)	P-value
Age (years)	28.03 ± 6.34	27.69 ± 9.45	28.60 ± 4.19	0.80
Sex				
Male	212 (36.8%)	75 (27%)	39 (46.4%)	0.001*
Female	364 (63.2%)	203 (73%)	45 (53.6%)	
Region				
Eastern	45 (7.8%)	31 (11.2%)	9 (10.7%)	0.98
Middle	123 (21.4%)	55 (19.8%)	19 (22.6%)	
Northern	43 (7.5%)	42 (15.1%)	17 (20.2%)	
Southern	120 (20.8%)	37 (13.3%)	11 (13.1%)	
Western	245 (42.5%)	113 (40.6%)	28 (33.3%)	
Marital status				
Married	201 (34.9%)	90 (32.4%)	14 (16.7%)	0.03*
Divorced	12 (2.1%)	3 (1.1%)	1 (1.2%)	
Widowed	1 (0.20%)	0	0	
Single	362 (62.8%)	185 (66.5%)	69 (82.1%)	
Education level				
Primary	3 (0.50%)	1 (0.40%)	1 (1.2%)	0.09
Secondary	10 (1.7%)	0	0	
High school	118 (20.5%)	74 (26.6%)	20 (23.8%)	
University/Above	445 (77.3%)	203 (73%)	63 (75%)	
Income (SAR)				
<1,000	231 (40.1%)	105 (37.8%)	26 (31%)	0.06
1,000–5,000	135 (23.4%)	67 (24.1%)	23 (27.4%)	
5,001–10,000	73 (12.7%)	32 (11.5%)	10 (11.9%)	
10,001–15,000	72 (12.5%)	38 (13.7%)	3 (3.6%)	
>15,000	65 (11.3%)	36 (12.9%)	22 (26.2%)	
Have you ever had a stroke before?	3 (7.5%)	2 (2.6%)	15 (1.8%)	0.06

Factors affecting the awareness of participants about stroke

Marital status and level of education were found to significantly affect the level of awareness, with females and subjects with secondary and university education having a high level of awareness (Table 4).

**Table 4:** Factors affecting awareness of participants about stroke. *: P-value is significant at <0.05. Data are presented as mean (SD) and frequency (percentage).

Item	Low (n = 40)	Mild (n = 78)	High (n = 820)	P-value
Age (years)	27.23 ± 6.98	29.11 ± 6.90	28.01 ± 4.67	0.56
Sex				
Male	20 (50%)	28 (35.9%)	278 (33.9%)	0.11
Female	20 (50%)	50 (64.1%)	542 (66.1%)	
Region				
Eastern	4 (10%)	6 (7.7%)	75 (9.1%)	0.70
Middle	6 (15%)	17 (21.8%)	174 (21.2%)	
Northern	1 (2.5%)	7 (9%)	94 (11.5%)	
Southern	9 (22.5%)	14 (17.9%)	145 (17.7%)	
Western	20 (50%)	34 (43.6%)	332 (40.5%)	
Marital status				
Married	19 (47.5%)	39 (50%)	247 (30.1%)	0.001*
Divorced	0	2 (2.6%)	14 (1.7%)	
Widowed	1 (2.5%)	0	0	
Single	20 (50%)	37 (47.4%)	559 (68.2%)	
Education level				
Primary	2 (5%)	1 (1.3%)	2 (0.20%)	<0.001*
Secondary	2 (5%)	3 (3.8%)	5 (0.6%)	
High school	7 (17.5%)	9 (11.5%)	196 (23.9%)	
University/Above	29 (72.5%)	65 (83.3%)	617 (75.2%)	
Income (SAR)				
<1,000	12 (30%)	21 (26.9%)	329 (40.1%)	0.06
1,000–5,000	9 (22.5%)	16 (20.5%)	200 (24.4%)	
5,001–10,000	6 (15%)	12 (15.4%)	97 (11.8%)	
10,001–15,000	8 (20%)	11 (14.1%)	94 (11.5%)	
>15,000	5 (12.5%)	18 (23.1%)	100 (12.2%)	
Have you ever had a stroke before?	3 (7.5%)	2 (2.6%)	15 (1.8%)	0.09

Factors affecting the knowledge of participants about the alarm signs of stroke

Sex and marital status significantly affected the level of knowledge of participants about alarm signs, with female and single subjects having a high level of knowledge about alarm signs (Table 5).

**Table 5:** Factors affecting knowledge of participants about alarm signs of stroke. *: P-value is significant at <0.05. Data are presented as mean (SD) and frequency (percentage).

Item	Low (n = 548)	Mild (n = 245)	High (n = 145)	P-value
Age (years)	29.11 ± 3.67	28.19 ± 6.11	27.90 ± 4.22	0.17
Sex				
Male	208 (38%)	73 (29.8%)	45 (31%)	0.04*
Female	340 (62%)	172 (70.2%)	100 (69%)	
Region				
Eastern	46 (8.4%)	20 (8.2%)	19 (13.1%)	0.17
Middle	106 (19.3%)	52 (21.2%)	39 (26.9%)	
Northern	67 (12.2%)	25 (10.2%)	10 (6.9%)	
Southern	105 (19.2%)	43 (17.6%)	20 (13.8%)	
Western	224 (40.9%)	105 (42.9%)	57 (39.3%)	
Marital status				
Married	198 (36.1%)	72 (29.4%)	35 (24.1%)	0.04*
Divorced	12 (2.2%)	3 (1.2%)	1 (0.7%)	
Widowed	1 (0.20%)	0	0	
Single	337 (61.5%)	170 (69.4%)	109 (75.2%)	
Education level				
Primary	4 (0.7%)	1 (0.4%)	0	0.39
Secondary	9 (1.6%)	1 (0.40%)	0	
High school	118 (21.5%)	60 (24.5%)	34 (23.4%)	
University/ above	417 (76.1%)	183 (74.7%)	111 (76.6%)	
Income (SAR)				
<1,000	216 (39.4%)	93 (38%)	53 (36.6%)	0.79
1,000–5,000	125 (22.5%)	63 (25.7%)	37 (25.5%)	
5,001–10,000	68 (12.4%)	34 (13.9%)	13 (9%)	
10,001–15,000	68 (12.4%)	26 (10.6%)	19 (13.1%)	
>15,000	71 (13%)	29 (11.8%)	23 (15.9%)	
Have you ever had a stroke before?	18 (3.3%)	2 (0.8%)	0	0.13

Factors affecting the knowledge of participants about complications of stroke

Sex and marital status significantly affected the level of knowledge of participants about complications of stroke, with female and single subjects having a high level of knowledge about complications of stroke (Table 6).

**Table 6:** Factors affecting knowledge of participants about complications of stroke. *: P-value is significant at <0.05. Data are presented as mean (SD) and frequency (percentage).

Item	Low (n = 633)	Mild (n = 212)	High (n = 93)	P-value
Age (years)	27.76 ± 4.40	28.22 ± 6.01	28.78 ± 6.19	0.45
Sex				
Male	229 (36.2%)	65 (30.7%)	32 (34.4%)	0.34
Female	404 (63.8%)	147 (69.3%)	61 (65.6%)	
Region				
Eastern	54 (8.5%)	19 (9%)	12 (12.9%)	0.06
Middle	136 (21.5%)	44 (20.8%)	17 (18.3%)	
Northern	58 (9.2%)	35 (16.5%)	9 (9.7%)	
Southern	126 (19.9%)	31 (14.6%)	11 (11.8%)	
Western	259 (40.9%)	83 (39.2%)	44 (47.3%)	
Marital status				
Married	227 (35.9%)	57 (26.9%)	21 (22.6%)	0.02*
Divorced	13 (2.1%)	3 (1.4%)	0	
Widowed	1 (0.20%)	0	0	
Single	392 (61.9%)	152 (71.7%)	72 (77.4%)	
Education level				
Primary	3 (0.50%)	2 (0.90%)	0	0.10
Secondary	10 (1.6%)	0	0	
High school	131 (20.7%)	59 (27.8%)	22 (23.7%)	
University/Above	489 (77.3%)	151 (71.2%)	71 (76.3%)	
Income (SAR)				
<1,000	243 (38.4%)	90 (42.5%)	29 (31.2%)	0.08
1,000–5,000	147 (23.2%)	47 (22.2%)	31 (33.3%)	
5,001–10,000	73 (11.5%)	31 (14.6%)	11 (11.8%)	
10,001–15,000	86 (13.6%)	21 (9.9%)	6 (6.5%)	
>15,000	84 (13.3%)	23 (10.8%)	16 (17.2%)	
Have you ever had a stroke before?	10 (1.6%)	10 (4.7%)	0	0.09

## Discussion

Because it causes severe impairment, functional limitations, and a decline in quality of life, stroke has a direct impact on health systems, imposing a significant financial burden and being considered a global public health concern. Stroke is one of the major and increasingly serious causes of sickness and death in Saudi Arabia [13].

Assessing the public’s understanding of stroke is crucial as it may help improve quality of life, limit complications, and prevent stroke development. Prior research has demonstrated that the Saudi populace lacks health literacy, which is linked to a lack of understanding of health information [15,16]. Thus, this study evaluated the Saudi population’s understanding of stroke, including its risk factors, early symptoms, and aftermath with different complications.

The average age of the enrolled subjects was 28.53 years. Most subjects were females (n = 612, 65.2%), and single (n = 616, 65.7%). Additionally, 711 (75.8%) participants had a university-level education or higher. Similarly, in the study of Alhubail et al., most participants were females (n = 285, 57%) and were aged <39 years (n = 333, 66.7%) [13].

General knowledge about stroke

Surprisingly, we found that only 84 (9%) participants had a high level of general awareness regarding stroke, and the majority (n = 576, 61.4%) had a low level of knowledge. Although most participants knew about the causes and risk factors of stroke, the knowledge score was low due to the low percentage of them knowing that epilepsy (n = 127, 13.5%), Alzheimer’s disease (n=279, 29.7%), and sun exposure (362, 38.6%) were not risk factors.

A similar study that tested the knowledge regarding stroke symptoms was conducted in Taif, Saudi Arabia, with a total of 3,456 individuals. In the study, only 300 (8.7%) were classified as having good knowledge, 746 (21.6%) as having fair knowledge, and 2,410 (69.7%) as having low information [17]. This is consistent with many global studies [18-20].

Nonetheless, a previous study demonstrated that most participants (82.6%) have adequate awareness of stroke symptoms [13]. A Lebanese study showed a highly satisfactory level of stroke symptom knowledge, with 93% of their study sample recognizing at least three right responses [21].

Awareness of participants about stroke

Fortunately, the majority (n = 820, 87.4%) had a high level of awareness. Furthermore, a high percentage knew that stroke is a medical emergency (n = 869, 92.6%), a cause of death (n = 872, 93%), and causes behavioral change (n = 895, 95.4%). In line with the current study, research done in 2011 in Saudi Arabia found that around 87% of people identified stroke as a medical emergency requiring immediate medical intervention [22].

Although there are many previously reported studies, the majority of participants had a good degree of knowledge about medical emergencies of stroke and its serious outcome [3,23]. Some studies found that most participants did not know that a stroke is a medical emergency and may cause death [12,19]. We thought that this discrepancy was mainly due to different characteristics of participants in different studies (age, level of education, sex).

Knowledge of alarm signs and complications

A low percentage of our participants had a high knowledge level about alarm signs (n = 145, 15.5%) and complications of stroke (n = 93, 9.9%). Regarding the level of knowledge about alarm signs of stroke, 548 (58.4%), 245 (26.1%), and 145 (15.5%) participants had low, mild, and high levels of knowledge, respectively. Regarding the level of knowledge about complications, 67.5%, 22.6%, and 9.9% of participants had low, mild, and high levels of knowledge, respectively.

The lower degree of knowledge of stroke warning indicators is consistent with previous reports [24-26]. Hickey et al. found warning indications were recognized by at least 5% of the research participants [26]. In contrast, approximately three-quarters of participants in a previously reported study knew about the alarm signs of stroke and considered dysarthria, which is difficulty in speaking or comprehending speech, as the most prevalent alarm sign of stroke, followed by sudden loss of consciousness [12]. This was consistent with previous studies [3,21].

We found that the most reported alarm signs of stroke were sudden confusion, difficulty speaking, or difficulty understanding speech (n = 670, 71.4%), followed by sudden numbness or paralysis of the face, arm, or leg (n = 647, 69%). In a previous study, up to 75% of participants believed dysarthria, or difficulty speaking or comprehending speech, to be the most prevalent indicator of stroke, followed by sudden loss of consciousness [13].

There was a wide variation in the reported most frequent alarm signs of stroke. According to some research, weakness or paralysis of one side of the body is the most prevalent indication of a stroke [21,23]. In other studies, numbness, weakness, and speech difficulties were identified as the most prevalent symptoms of stroke [27,28]. In Australia, participants reported blurred, double, or loss of vision as the most common early stroke symptoms [28].

In our study, we found that 639 (68.1%), 577 (61.5%), and 622 (66.3%) of participants considered that paralysis, cerebral edema, and memory loss are complications of stroke. Similarly, in a previous study, 381 (76.2%) individuals felt that suffering a stroke would result in lasting impairment and memory loss [13].

Factors affecting the level of knowledge and awareness

Generally, females with a high level of education and single subjects had a better level of knowledge and awareness. Female and single subjects had a high level of knowledge. Moreover, females with secondary and university-level education had a high level of awareness. At the same time, female and single subjects had a high level of knowledge about alarm signs.

Previous research indicated that higher education and employment had a significant impact on participants’ knowledge and awareness of stroke [3,13,21,29]. In a Saudi study, individuals with higher educational levels, full-time jobs, and medical backgrounds had a better understanding of stroke risk factors and early symptoms [12]. This is to be expected as more education typically leads to greater job prospects and, consequently, higher incomes, as well as improved access to health services and health insurance, which facilitate people’s ability to obtain and comprehend health information.

Limitations

There are various limitations of this study. Because the majority were women with high levels of education, the findings cannot be deemed typical of the Saudi community. Furthermore, causality cannot be deduced from a cross-sectional analysis. Because the study’s questionnaire was self-reported and conducted online, information bias may be present. Because the sample was acquired via the snowball sampling technique rather than being chosen at random, selection bias may have occurred. Because this study did not examine all the characteristics associated with stroke awareness, residual confounding bias may exist. Additionally, we simply evaluated the degree of awareness regarding stroke and did not confirm the knowledge scale. Furthermore, we based our research on other studies to identify a particular stroke symptom or risk factor.

Despite these limitations, our study adds data to the literature on stroke illness awareness, which emphasizes the Saudi population’s lack of understanding of stroke. Additionally, we conducted this survey in all regions of the Kingdom, improving the generalizability of the findings.

## Conclusions

Despite widespread awareness of stroke as a medical emergency, the Saudi population still has considerable gaps in their understanding of stroke risk factors, early symptoms, and sequelae. These disparities are most noticeable for married people, men, and those with less schooling. To improve outcomes and prevent stroke, public health efforts should concentrate on culturally appropriate educational interventions that address these inadequacies, with a particular emphasis on symptom assessment and risk management.

## Appendices

Please visit the link to access the study questionnaire: https://docs.google.com/forms/d/e/1FAIpQLSeCfT1EegHqFMo0ZaiGcFAAt0RE3xCvG43ZDvItbdEfyKjQOA/viewform.
